# Combination therapy including dupilumab, omalizumab, and mycophenolate mofetil for refractory bullous pemphigoid

**DOI:** 10.1016/j.jdcr.2025.08.001

**Published:** 2025-08-19

**Authors:** Kayadri Ratnarajah, Steffie Arès, Elio Kechichian, Carolina Lucena Fernandes

**Affiliations:** a Division of Dermatology, McGill University Health Center, Montreal, Quebec, Canada; b Faculty of Medicine, Université de Sherbrooke, Sherbrooke, Quebec, Canada; c Division of Dermatology, Université de Sherbrooke, Sherbrooke, Quebec, Canada

**Keywords:** autoimmune blistering diseases, bullous pemphigoid, combination therapy, dupilumab, omalizumab

## Background

Bullous pemphigoid (BP) is a chronic, autoimmune blistering disorder that requires effective management, especially in severe cases or those with contraindications to conventional therapies. The combination of dupilumab and omalizumab (OMZ) has been explored in these challenging scenarios.^1^, ^2^, ^3^, ^4^, ^5^

### Case 1

A 54-year-old male with a history of psoriasis and recent cardiac surgeries for aortic dissection presented with erythematous, pruritic plaques and bullae on his extremities and trunk. Histopathology confirmed subepidermal cleavage with eosinophilic and lymphocytic infiltrates. Direct immunofluorescence revealed linear IgG and C3 deposits along the basement membrane zone (BMZ). A salt-split skin test was positive on the blister roof. Serum eosinophils were elevated (1.5 × 10^9^/L). Indirect immunofluorescence was not available during diagnosis.

Initially, the patient was treated with potent topical corticosteroids, which failed to provide relief. Methotrexate (MTX) at 17 mg weekly was initiated, with prednisone 25 mg daily, and doxycycline was later added. Despite escalation of MTX to 25 mg, flares recurred with tapering of prednisone. After 7 months without full remission, OMZ (300 mg subcutaneously every 4 weeks) was introduced and later increased to every 2 weeks. While partial improvement was noted after 2 months on OMZ and MTX, the patient remained highly symptomatic.

Given the refractory nature of the disease, MTX was replaced by mycophenolate mofetil (MMF) at 2.5 g daily. The number of lesions decreased significantly, but the patient still suffered from severe pruritus, developing 20 new lesions per week. After 1 year of treatment with MMF and OMZ, the addition of dupilumab (300 mg every 2 weeks) was introduced. After 7 months of combined therapy, the patient achieved disease control, allowing for successful prednisone tapering. During this time, multiple attempts to discontinue MMF were unsuccessful.

Repeated attempts to taper MMF resulted in disease relapse. The patient maintained remission for over 18 months before accidental death. The combination was well tolerated, with no adverse effects.

### Case 2

A 59-year-old male with type 2 diabetes mellitus, end-stage renal disease on dialysis, and active prostate adenocarcinoma presented with generalized erythematous pruritic plaques and tense bullae during hospitalization for severe renal failure and respiratory insufficiency. He was on tacrolimus and prednisone 5 mg/day for a previous renal transplant. A skin biopsy revealed cleavage between the dermis and epidermis, with eosinophilic infiltrates and polymorphonuclear eosinophils and neutrophils. Direct immunofluorescence confirmed linear IgG and C3 deposits along the BMZ, and the salt-split skin test was positive at the blister roof. Indirect immunofluorescence was also positive for epidermal BMZ antibodies. Serum eosinophils were elevated (2.4 × 10^9^/L).

Due to the patient's multiple comorbidities and limited immunosuppressive options, prednisone (50 mg daily), doxycycline, and OMZ (300 mg every 4 weeks) were started. After 7 months of treatment, the patient achieved partial disease control, and poor medication compliance triggered an exacerbation. Prednisone was increased, and the frequency of OMZ was shortened to every 2 weeks. Despite this, bullae and pruritus persisted. A low dose of MMF (1 g/day) was added with nephrology approval, but its efficacy was limited due to dosage restrictions. Intravenous immunoglobulin therapy was contraindicated by the nephrology team due to the risk of decreasing the patient’s residual diuresis. Due to end-stage renal disease in a previously transplanted patient and the presence of active prostate cancer, the addition of a systemic immunosuppressant had to be minimized.

In response to ongoing flare-ups, dupilumab (300 mg every 2 weeks) was introduced as bridge therapy for 8 weeks, resulting in clearance of the disease and successful prednisone tapering. After 7 months of remission, a flare occurred, likely due to missed doses of MMF. Dupilumab was reintroduced for another 8-week course, again achieving disease control. Both drugs were well tolerated, and the combination provided effective treatment even when conventional therapies failed. The patient remained in remission for over a year, until death from renal complications.

## Discussion

BP is an autoimmune blistering disorder characterized by autoantibodies targeting hemidesmosomal proteins, particularly BP180 and BP230, critical for dermal-epidermal junction adhesion.^3^^,^^6^^,^^7^ A Th2-mediated inflammatory response, marked by IL-4 and IL-13, promotes autoantibody production and eosinophil recruitment, exacerbating blister formation and chronic pruritus.^5^^,^^8^, ^9^, ^10^

**Fig 1:**
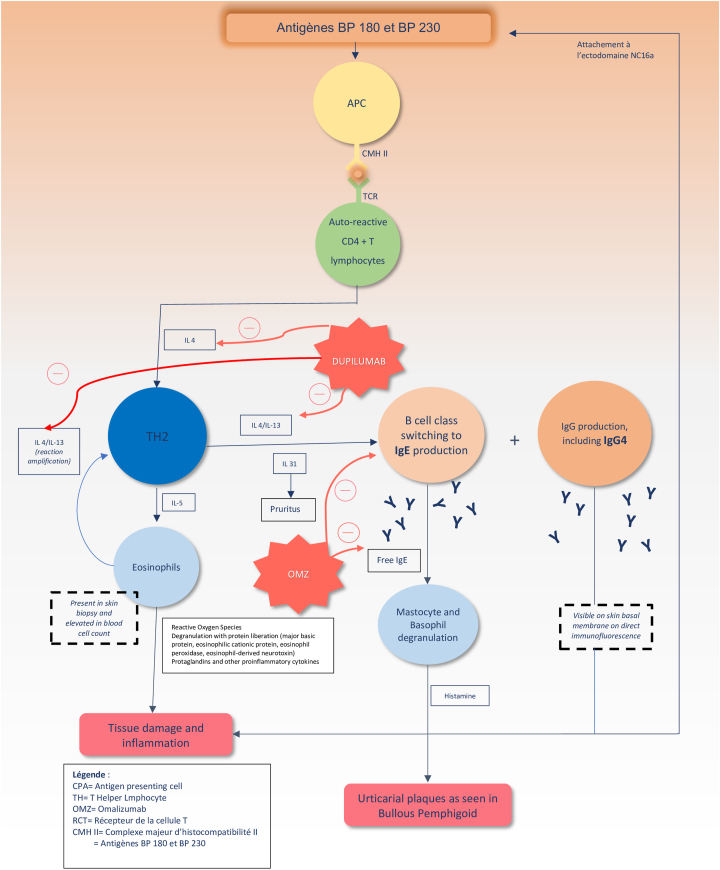
Pathogenesis of bullous pemphigoid and targets of dupilumab and OMZ. Bullous pemphigoid is initiated by an autoimmune response against BP180 and BP230 antigens. APCs present these antigens via MHC class II to autoreactive CD4+ T cells, leading to a TH2-skewed immune response with production of IL-4, IL-5, IL-13, and IL-31. This promotes class-switching of B cells to IgE and IgG4 production and recruitment of eosinophils and mast cells. Eosinophils contribute to tissue damage via the release of cytotoxic granules and reactive oxygen species. Mast cell and basophil degranulation result in histamine release, contributing to pruritus and urticarial plaques. Dupilumab, an IL-4Rα antagonist, inhibits IL-4 and IL-13 signaling, thereby attenuating TH2 polarization, IgE class switching, and pruritus. OMZ, an anti-IgE monoclonal antibody, blocks IgE-mediated mast cell activation. This figure highlights the overlapping and complementary mechanisms of dupilumab and OMZ in disrupting the TH2-driven pathogenesis of BP. *APC*, Antigen-presenting cell; *IgE*, immunoglobulin E; *IgG4*, immunoglobulin G4; *IL*, interleukin; *IL-4Rα*, interleukin 4 receptor alpha chain; *MHC II*, major histocompatibility complex class II; *OMZ*, omalizumab; *ROS*, reactive oxygen species; *TCR*, T-cell receptor; *TH2*, T helper 2 lymphocyte.

Dupilumab, initially approved for atopic dermatitis and asthma, blocks IL-4 and IL-13 signaling and has shown promising results in BP, especially in severe cases or when traditional therapies fail. OMZ, an anti-immunoglobulin E (IgE) monoclonal antibody, reduces IgE levels and mast cell activation, benefiting BP patients, particularly those with elevated IgE^3^^,^^5^^,^^10^, ^11^, ^12^, ^13^, ^14^, ^15^, ^16^ (Fig 1).

Both therapies show safety and efficacy in BP, with our cases highlighting their synergistic potential (Table I). Dupilumab and OMZ are particularly beneficial in severe, refractory BP cases, especially in patients with comorbidities or contraindications to traditional immunosuppressive treatments. This combination therapy can act as a rescue treatment during exacerbations and is key to achieving long-term disease control, aiding in prednisone tapering. Our findings suggest that combining these biologics offers a promising alternative for patients with limited treatment options. While the relative contribution of each agent in the combination regimen remains uncertain, our cases highlight the potential safety and feasibility of combining dupilumab, OMZ, and MMF in treatment-refractory patients.

**Table I:** Patient characteristics and clinical response to dupilumab and omalizumab

Characteristics	Patient 1	Patient 2
Age/sex	54/Male	59/Male
Race	Caucasian	Caucasian
Lesion distribution	Extensive cutaneous	Extensive cutaneous + mucosa
Initial BPDAI score	103	182
Diagnostic method	H&E, DIF, salt-split	H&E, DIF, salt-split, indirect immunofluorescence
Ineffective or contraindicated treatments	Ineffective: MTX	Contraindications: High doses of immunosuppressants IVIg
Concomitant medication received with dupilumab treatment	MMF (2.5 g BID) OMZ Doxycycline	MMF (1 g daily maximal permitted dose) OMZ
Dupilumab treatment type	COMBINATION therapy	RESCUE therapy
Dose of dupilumab	300 mg subcutaneously every 1 to 2 weeks	300 mg subcutaneously every 2 wk
Dose of omalizumab	300 mg subcutaneously every 2 to 4 wk	300 mg subcutaneously every 2 to 4 wk
Duration of treatment (mos)	10	6
Duration of remission (mos)	More than 18 mo	More than a year
Response to dupilumab in combination with omalizumab	Disease clearance∗	Disease clearance∗
Prednisone doses	25 mg per day, tapered after more than 2 y	1 mg/kg per day (50 mg), tapered over several weeks
Successful prednisone tapering	Yes	Yes
Follow-up	On remission by the time of death	On remission by the time of death
Cause of death	Car crash	Complications related to kidney failure

*BPDAI*, Bullous Pemphigoid Disease Area Index; *H&E*, hematoxylin and eosin stain; *IVIg*, intravenous immunoglobulin; *MMF*, mycophenolate mofetil; *MTX*, methotrexate; *OMZ*, omalizumab.

∗ Defined as clearance of bullae and pruritus.

Rituximab, a third-line treatment for BP, has demonstrated response rates up to 75% but was not attempted in our patients due to severe comorbidities.^17^^,^^18^ Our first patient had recently undergone cardiac surgery for aortic dissection, with minimal immunosuppression advised. Our second patient, already on tacrolimus and prednisone for a renal transplant and with active prostate cancer, required avoidance of additional immunosuppressive therapy. A systematic review showed similar clinical improvement with rituximab, OMZ, and dupilumab, but rituximab had a higher recurrence rate and associated infection risks. A retrospective review of rituximab and OMZ in BP demonstrated a 70% response rate, but given rituximab’s prolonged immunosuppressive effects, it was deemed unsuitable for our patients with severe comorbidities.^17^

Few studies have evaluated the combination of dupilumab and OMZ for BP, with only 2 prior cases reported. Both showed complete disease clearance without adverse effects, underscoring the need for more research to assess the efficacy and safety of this combination in larger populations.^17^^,^^19^

Combination therapy with dupilumab and OMZ can be cost-effective, but securing insurance coverage for 2 biologics remains challenging, particularly in the United States.

## Conclusion

These cases demonstrate the efficacy and tolerability of dupilumab, OMZ, and MMF combination therapy for severe BP. This combined approach proves valuable for refractory cases and as continuous therapy for patients with contraindications or first-line treatment failure. Given the favorable safety profiles of these agents, further studies are needed to explore the broader cost-effectiveness and long-term outcomes of this therapeutic strategy in BP management.

## Conflicts of interest

None disclosed.
